# Implications of base cation deposition from increased climate-driven wildfires in the United States

**DOI:** 10.21203/rs.3.rs-10670869/v1

**Published:** 2026-09-07

**Authors:** Patrick Campbell, Daniel Tong, Shinkuang Chang, Siqi Ma, Yunyao Li, Jiaxin Ding, Rick Saylor, Barry Baker, Youhua Tang, John Walker

**Affiliations:** George Mason University; George Mason University; George Mason University; George Mason University; The University of Texas at Arlington; George Mason University; George Mason University; National Oceanic and Atmospheric Administration; George Mason University; United States Department of Agriculture

**Keywords:** Wildfire deposition, Base cations, Reactive nitrogen (Nr), Critical loads (CLs), Atmospheric chemistry modeling

## Abstract

Climate-driven increases in wildfires are altering atmospheric deposition patterns across the United States, with implications for ecosystem acidification and critical load (CL) exceedances. Using the 2002–2021 North American Chemical Reanalysis (NACR), we quantify multi-decadal trends in fire-derived base cation (Ca^2+^, Mg^2+^, K^+^, Na^+^) deposition and evaluate their capacity to buffer concurrent increases in reactive nitrogen (Nr) and sulfur (S) deposition from fires. We find substantial fire-derived enhancements in base cation deposition, often exceeding 100% near major wildfire regions, yet these increases are spatially inconsistent with Nr and S deposition maxima. Our results suggest that regions exhibiting the greatest relative increases in base cation deposition do not regularly coincide with those experiencing the largest Nr increases, thus limiting the potential for widespread acid buffering. After 2017, base cation contributions from fires rise sharply (~ 30 to 80%), reflecting intensifying fire activity, but combined Nr + S deposition remains overwhelmingly dominant, with increases 100 times larger than base cations due to fires. Overall, our results show that although fire-derived base cations provide localized buffering, they do not offset the broader rise in acidifying Nr and S deposition driven by climate-driven wildfires, reinforcing concerns about increasing CL exceedances in vulnerable western U.S. ecosystems.

## Introduction

Research has demonstrated the importance of atmospheric deposition of base cations (Ca^2+^, Mg^2+^, K^+^, Na^+^) in buffering soil acidity and influencing critical loads (CLs) for reactive nitrogen (Nr) deposition across different land and vegetative ecosystems (Whitfield et al., 2009; Watmough et al., 2014). Wildfires and prescribed burns can drive significant short-term increases in the combustion of surface organic matter, which may result in white ash formation when surficial organic layers are completely burned (Ulery et al., 2017). When emitted into the atmosphere, base cations are transported downwind and deposited onto forested ecosystems, where they can partially offset the acidifying effects of Nr deposition (Fenn et al., 2015).

Campbell et al. (2026) analyzed the 2002–2021 North American Chemical Reanalysis (NACR) dataset (Campbell et al., 2025) and showed that increased climate-driven wildfires have led to increases in emissions and deposition of Nr and can contribute to potential exceedances in CLs downwind by up to 20–40% in the western U.S. However, the NACR dataset contains additional chemical information on the emission, formation, transport, and fate (both wet and dry deposition) for all major organic, inorganic, and ion species, including base cation-bearing particulate matter.

Given the simultaneous emissions of particulate base cations and acid-forming Nr from fires, areas of increased Nr deposition downwind may be coincidently impacted by the buffering effects of alkaline-induced particulate deposition, thus potentially offsetting the reported increase in potential CL exceedances. Thus, the main objectives in this study are to expand upon Campbell et al. (2026) and analyze the multi-decadal NACR spatiotemporal trends of climate-driven increases in wildfires, downwind deposition of Nr, acidic sulfur, and base cations.

## Results

### Contributions of base cation deposition from fires

Here we expand upon Campbell et al. (2026) and provide additional analyses on the role of fire-derived base cation (particulate Ca^2+^, Mg^2+^, K^+^, Na^+^) deposition and its implications for increased Nr deposition and CL assessments. Our analysis considers base cation emissions associated with the fires themselves, rather than combined fire and dust emissions from the post-fire landscape (Wagenbrenner et al., 2017). Analysis of NACR simulations averaged over 2002–2021, including baseline (no-fire), absolute fire-attributed changes, and relative fire-attributed changes, shows substantial increases in total base cation deposition in regions near fire sources and downwind, particularly in western U.S. wildfire regions where relative increases can exceed 100% (Fig. 1a-c).

However, comparison of the ratio of relative changes in Nr and base cation deposition reveals distinct differences in their spatial patterns. Regions exhibiting the greatest relative increases in base cation deposition do not regularly coincide with those experiencing the largest Nr increases, especially in the west-northwestern United States (Fig. 1d). Faster dry deposition rates of reduced NH species, particularly NH_3_, dominate total Nr deposition downwind of fires, resulting in deposition maxima closer to emission sources compared to base cations, which tend to deposit farther downwind. These contrasting deposition patterns are illustrated by regions shaded in red (Nr-dominated) and blue (base cation–dominated) in Fig. 1d.

These results indicate that while fire-derived base cation deposition can buffer the acidifying effects of Nr and reduce potential CL exceedances in some regions, distinct areas of the western United States remain vulnerable to these detrimental effects. In these areas, increased Nr deposition associated with fires may still lead to elevated CL exceedances in the 2002–2021 average and trends, as was shown and discussed in Campbell et al. (2026).

### Increasing base cation deposition trends from fires

Analysis of annual total (wet + dry) base cation (Ca^2+^, Mg^2+^, K^+^, Na^+^) deposition trends show statistically significant increases in the relative percent contribution attributable to fires (Mann–Kendall ≈ 0.77–0.98), particularly after 2017 (Fig. 2).

In western U.S. regions, relative increases reach 30–80% after 2017 (Fig. 2b). Prior to 2017, trends are comparatively flat, indicating minimal fire-driven changes in base cation deposition during earlier years.

### Roles of increasing sulfur deposition from fires

Further analysis shows the impacts of acidic sulfur (S = SO_2_ + sulfuric acid gas + particulate sulfate ion) deposition trends and detrimental impacts on ecosystems and CLs (Clark et al., 2018; Rickly et al., 2022). Like Nr, fires have been shown to enhance S emissions and downwind deposition, particularly in the northwestern U.S. (Fig. 3); however, relative increases in S deposition due to fires is lower compared to Nr (see Fig. 2 of Campbell et al., 2026). Comparison of the ratio of relative changes in S and base cation deposition reveals similar shifts (i.e., ratios closer to or < 1; Fig. 3d), but there are still distinct differences in spatial patterns in the northwest U.S.

## Discussion

Placing our work into further context with past observations, Karlsson et al. (2013) found that high NH_4_^+^ dry deposition in a forest in Sweden was associated with a smoke plume from fires in Eastern Europe. Furthermore, Lill et al. (2026) showed that wildfire smoke in 2023 also led to increases in NH_4_^+^ deposition flux at all National Atmospheric Deposition Program (NADP) monitoring sites in the Upper Midwest. Olson et al. (2023) showed higher concentrations of base cations in PM_2.5_ associated with smoke in the western US.

As discussed in Campbell et al. (2026), there remains uncertainty in the NACR model estimates of Nr deposition rates, especially for dry deposition. This is true here as well for S and base cations, particularly in the formulations for SO_2_ dry deposition rates. It has been recently shown that concurrent alkaline dust deposition can enhance dry deposition velocity for SO_2_, and that pH-modulated dry deposition to foliage surfaces can improve modeled SO_2_ concentrations in these regions (Miller et al., 2026). As forest fire emissions result in relatively elevated emissions of concurrent alkaline base cations and SO_2_ (e.g., regions with S to base cation ratio near 1 in Fig. 3d), neglecting this effect here in the NACR simulations suggests that the SO_2_ dry deposition rates may be underestimated in some areas.

In summary, here we provide further information on the role of increased fires and base cation deposition in context to our previous work on Nr deposition (Campbell et al., 2026). Our previous work (Campbell et al. 2026) showed increasing trends in climate-driven fires and emissions, particularly from wildfires in the western U.S. between 2002–2021. Expanding upon Campbell et al. here shows that the enhanced trends in climate-driven fires lead to increased Nr and S deposition and potential critical load exceedances to downwind ecosystems, despite coincident (but lower and spatially inconsistent) increases in base cation deposition (Fig. 4). Therefore, these results further support the main findings of Campbell et al. (2026).

## Methods

As described in full detail in Campbell et al. (2026), this study uses the North American Chemical Reanalysis (NACR) system to examine long-term (2002–2021) meteorological conditions, air quality, and nitrogen (Nr), sulfur (SO_2_ + particulate sulfate), and base cation (particulate Ca^2+^, Mg^2+^, K^+^, Na^+^) deposition across the U.S., with a focus on wildfire impacts. NACR combines the Weather Research and Forecast (WRF) meteorological model, downscaled from ERA5, and the Community Multiscale Air Quality (CMAQ) model with detailed emissions from the EPA’s Air QUAlity TimE Series Project (EQUATES) for 2002–2017 and National Emissions Inventory (NEI) for 2018–2021. Two simulations (with and without fire emissions) quantify fire contributions to Nr, S, and base cation deposition.

The NACR system incorporates advanced physics, chemistry, aerosol, and deposition schemes, and processes anthropogenic and fire emissions through the Sparse Matrix Operator Kernel Emissions (SMOKE) processing system, with biogenic and sea-salt emissions calculated inline. Trend analyses use Mann-Kendall and Theil-Sen methods, and results are aggregated by NOAA climate regions.

Model evaluations in Campbell et al. (2026) show that NACR captures long-term trends in meteorology, gases, and particulates, with improved performance when fire emissions are included. However, biases persist, such as underpredicted NH_3_ and wet deposition and overpredicted HNO_3_ and NO_3_^−^ dry deposition, stemming from uncertainties in emissions, deposition algorithms, and precipitation. Despite these limitations, NACR effectively characterizes spatial and temporal Nr deposition patterns and highlights wildfire contributions, especially in the western U.S.

## Figures and Tables

**Figure 1.**
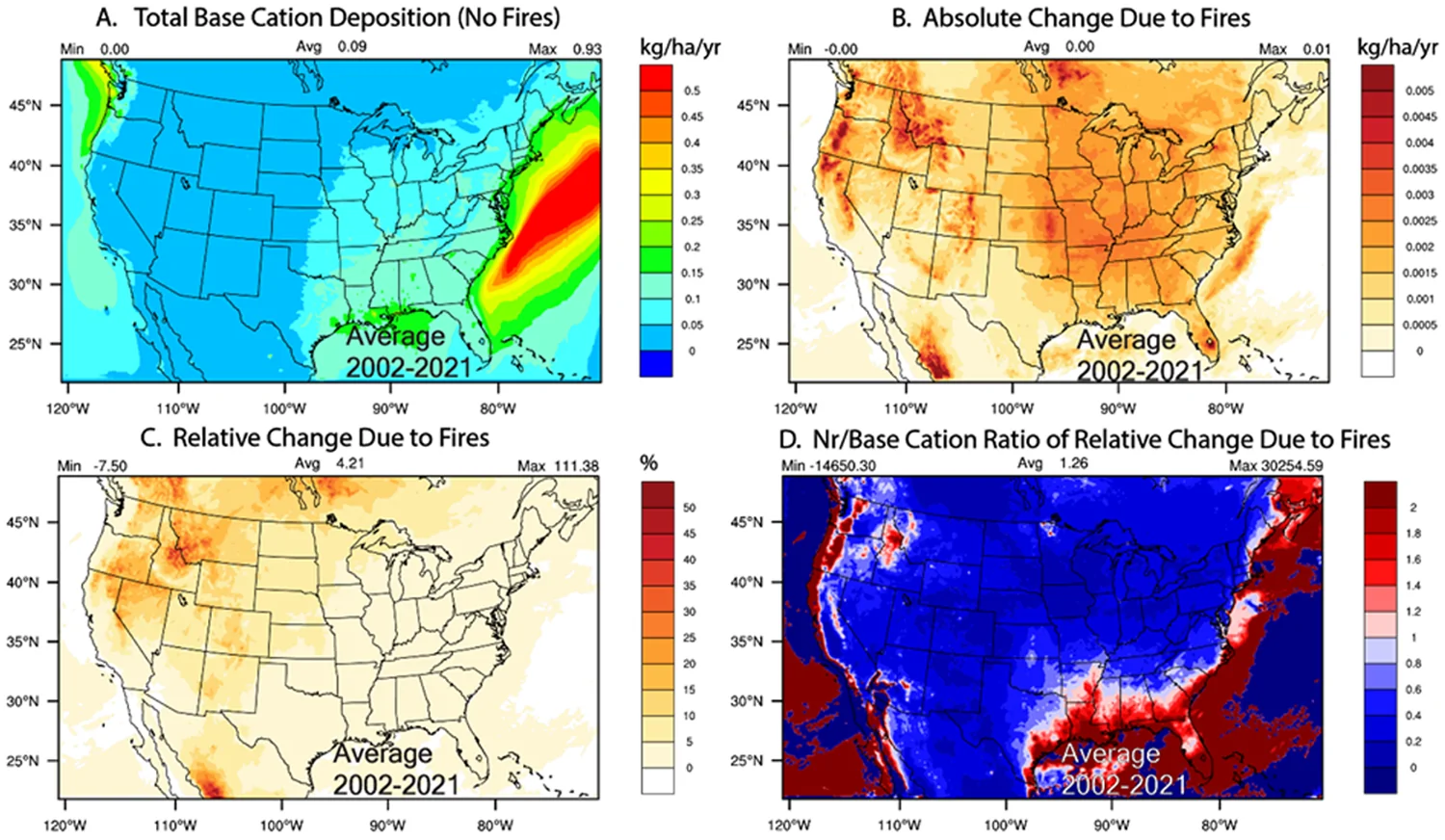
Spatial Patterns of Annual Total Base Cation Deposition Annual 2002 – 2021 average total base cation (Ca^2+^, Mg^2+^, K^+^, Na^+^) deposition for a) absolute (kg/ha/yr) total (wet + dry), b) absolute (kg/ha/yr) difference (FIRE-NOFIRE), c) relative (%) difference (FIRE-NOFIRE/NOFIRE), and d) ratio of Nr to base cation relative difference (red = Nr > base cation, blue = Nr < base cation). Note a caveat of our study is that crustal elements (Ca^2+^, Mg^2+^, Fe^2+^) are underestimated in the southwest U.S. due to missing windblown dust emissions

**Figure 2.**
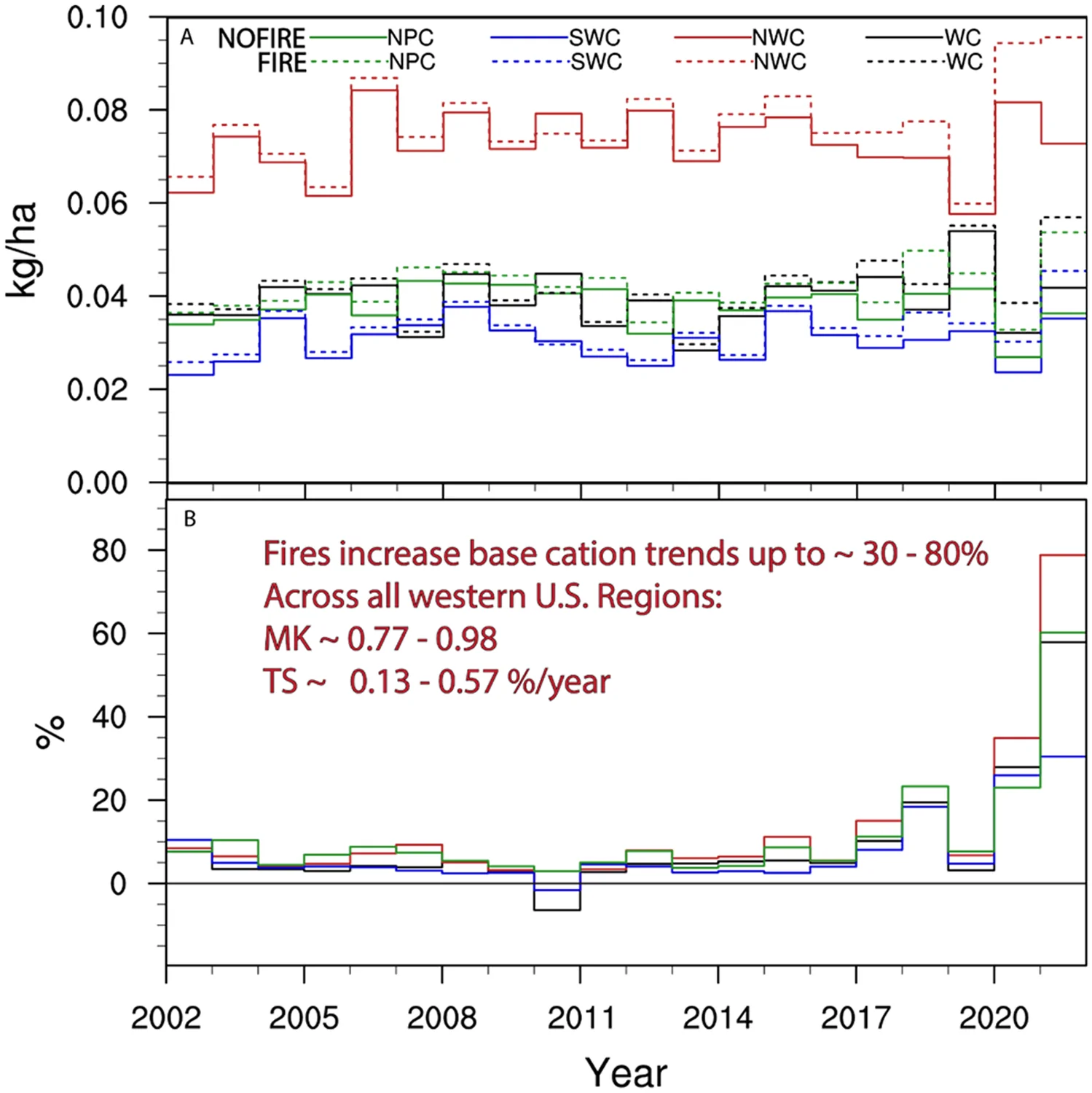
Trends in the Annual Total Base Cation Deposition and Contributions from Fires Annual 2002 – 2021 total (wet + dry) a) base cation (Ca^2+^, Mg^2+^, K^+^, Na^+^) deposition for the NOFIRE (solid lines) and FIRE runs (dashed lines) and b) relative difference ([[FIRE-NOFIRE]/NOFIRE] * 100 %)) averaged across each western U.S. fire climate region (see Supporting Fig. S2 in Campbell et al. (2026) for definition of the NPC, SWC, NWC, and WC regions). Panel B includes relative (%) difference (FIRE-NOFIRE/NOFIRE) for Nr/CL trends, where insert values pertain to Mann-Kendall (MK) scores and Theil-Sen (TS) trends across western U.S.

**Figure 3.**
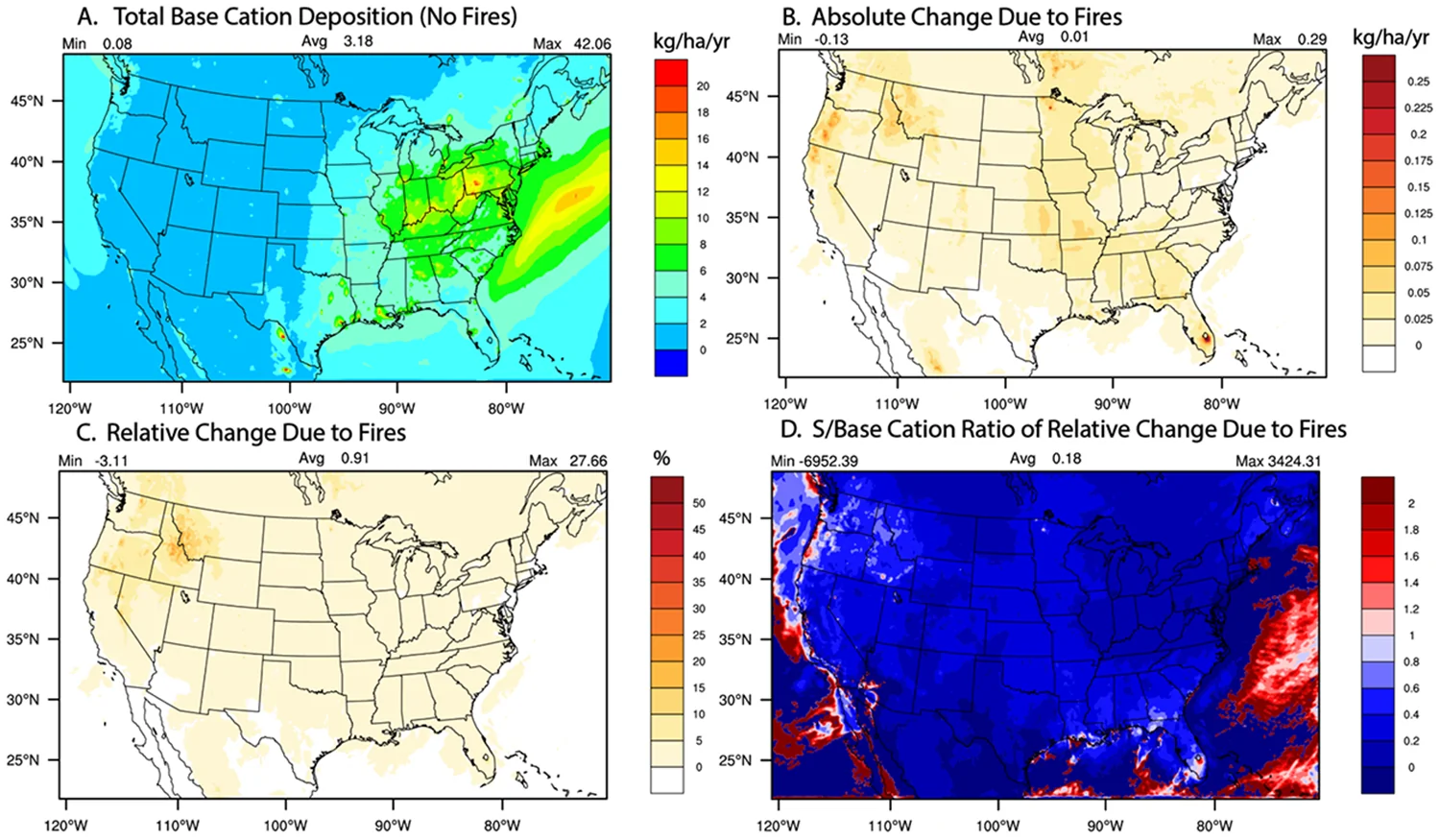
Spatial Patterns of Annual Total S Deposition Annual 2002 – 2021 average total S (SO_2_ + sulfuric acid gas + particulate sulfate ion) deposition for a) absolute (kg/ha/yr) total (wet + dry), b) absolute (kg/ha/yr) difference (FIRE-NOFIRE), c) relative (%) difference (FIRE-NOFIRE/NOFIRE), and d) ratio of S to base cation relative difference (red = S > base cation, blue = S < base cation).

**Figure 4.**
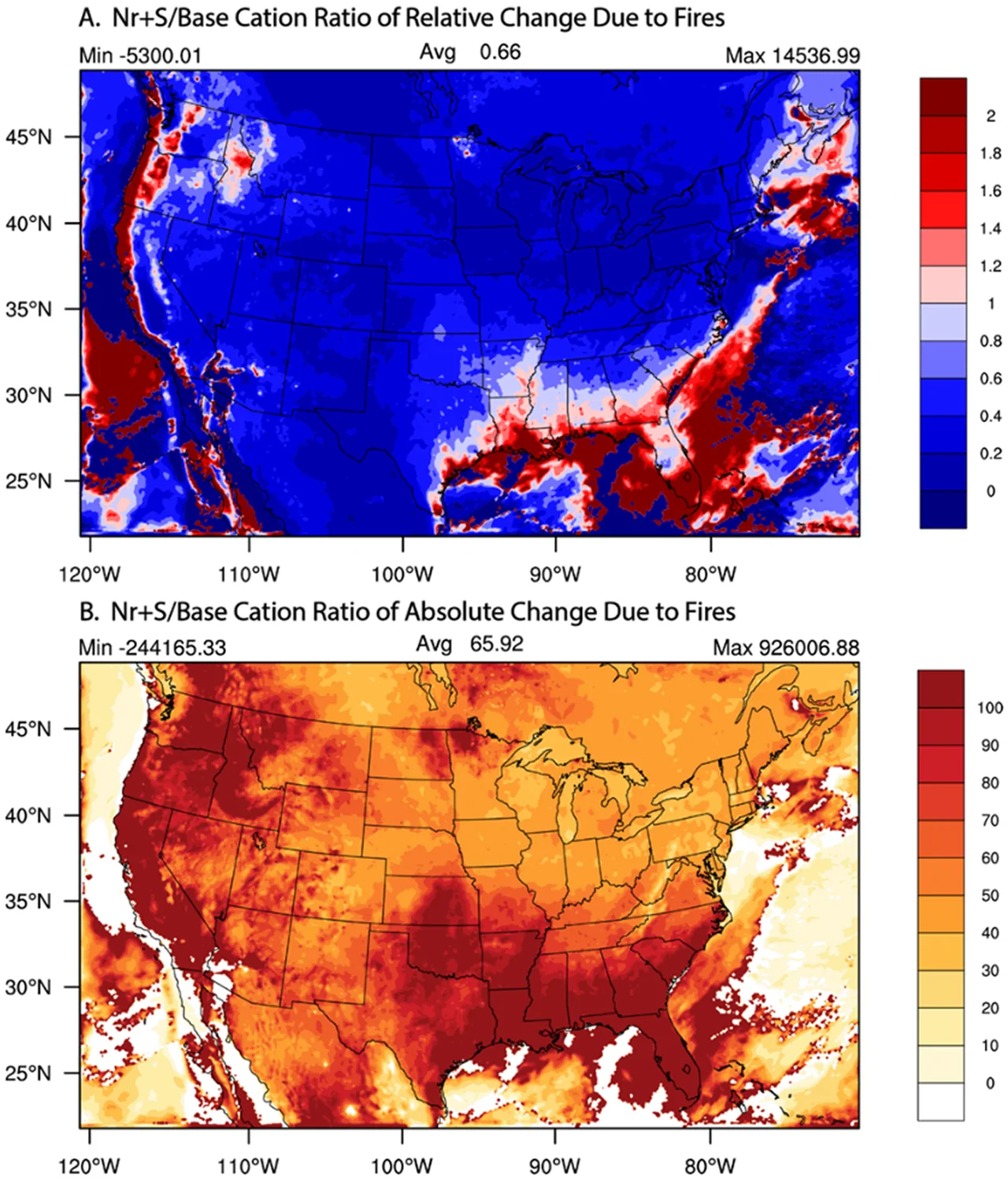
Spatial Patterns of Annual Ratio of Total Nr+S Deposition to Base Cations Annual 2002–2021 average deposition (wet+dry) for a) ratio of Nr+S/base cations for relative changes due to fires and b) ratio of Nr+S/base cations for absolute changes due to fires.

## Data Availability

All NACR 2002-2021 post-processed data and scripts used in this paper for land, meteorology, emissions, and chemistry/deposition analyses are publicly available online at: https://zenodo.org/records/21458453 The EQUATES dataset is available for download from the Community Modeling and Analysis System (CMAS) Data Warehouse available online at: https://www.epa.gov/cmaq/equates.

## Code Availability

WRFv4.2: https://github.com/wrf-model/WRF/releases/tag/v4.2

CMAQv5.3.3: https://doi.org/10.5281/zenodo.5213949

SMOKE: https://www.cmascenter.org/smoke/
